# Epidemiology and Outcomes from Out-of-Hospital Cardiac Arrest in Kuwait

**DOI:** 10.1155/2020/9861798

**Published:** 2020-04-23

**Authors:** Dalal Al Hasan, Ameen Yaseen, Mazen El Sayed

**Affiliations:** ^1^Department of Applied Medical Sciences, Health Sciences College, Public Authority of Applied Education and Training, Adailiyah, Kuwait; ^2^Audit Department, Emergency Medicals Services, Adailiyah, Kuwait; ^3^Department of Emergency Medicine, American University of Beirut Medical Center, Beirut, Lebanon

## Abstract

**Background:**

Out-of-hospital cardiac arrest (OHCA) survival remains low in most countries. Few studies examine OHCA outcomes out of the Middle East region. This is the first study to describe characteristics and outcomes of patients with OHCA treated by emergency medical services (EMS) in regions of Kuwait.

**Objectives:**

To describe characteristics and outcomes of adult patients affected with OHCA in regions of Kuwait.

**Methods:**

This was a retrospective observational study on all adult OHCA patients transported by EMS to regional emergency departments over a 10- month period (21 February–31 December 2017). Data were collected from various sources: national emergency medical services archived data, emergency department, intensive care unit, and cardiac care unit of two hospitals.

**Results:**

A total of 332 EMS-treated OHCA cases were reviewed, and 286 incidents with OHCA from cardiac aetiology were included in the study. Most were non-Kuwaiti (60.8%) males (67.1%) with mean age 61 (+−16) years. Most OHCA cases occurred at home (76%) but with low witness rate (11.5%). Bystander CPR rate was low (8.7%). ROSC was achieved in ten patients (3.5%), but only 1 (0.3%) patient survived to hospital discharge.

**Conclusion:**

OHCA survival rates in this region of Kuwait are low. Targeted measures such as creating cardiac registry, dispatcher-assisted CPR with ongoing training and quality improvement, and community-based CPR education program are needed to improve the survival rates of OHCA victims.

## 1. Introduction

Out-of-hospital cardiac arrest survival rates in the Middle East remain low compared to those in North America and Europe [1, 2]. Recent studies confirmed unique demographics and low OHCA survival rates in the Middle Eastern region [3, 4]. Poor survival rates are usually related to lack of structural elements, processes, or evidence-based practices that ensure a comprehensive approach to care for OHCA victims from different stakeholders involved including EMS, community, and hospitals [5, 6].

One approach to improve community OHCA survival is emergency medical services (EMS) adherence to global resuscitation alliance recommendation on best practices including the following: establish a cardiac arrest registry; begin telephone-CPR with ongoing training and quality improvement (QI); begin high-performance EMS cardiopulmonary resuscitation (CPR) with ongoing training and QI begin; rapid dispatch; measure professional resuscitation using the defibrillator recording; begin an automated external defibrillation (AED) program for first responders, including police officers and other security personnel; use smart technologies to extend CPR and public access defibrillation programs; make CPR and AED training mandatory in schools and the community; work toward accountability; and work toward a culture of excellence [7]. Steady efforts in achieving best practices in these ten programs should improve OHCA outcomes [7].

To date, Middle Eastern health care systems have not shown progress in improving OHCA recognition, management, and outcomes. Baseline studies examining OHCA outcomes revealed poor survival rates in Beirut [8] and Riyadh [4]. OHCA survival has not been reported yet out of Kuwait. The objective of this study is to describe characteristics and outcomes of adult patients affected with OHCA in Hawali and AL-Farwanya provinces. It also aims at the development and initiation of OHCA chain of survival in Kuwait.

## 2. Method

### 2.1. Setting and Design

The study was conducted in two large provinces in Kuwait. Kuwait has a Ministry of Health's two-tiered EMS system that serves estimated 4,132,400 people [9] covering Kuwait's six provinces: Al-Asimah, Hawali, Al-Farwanya, Mubarak Al-Kabeer, Al-Jahra, and Al-Ahmidi. Hawali province is a heterogeneous urban area with 192,778 Kuwaitis and 480,132 non-Kuwaitis. Al-Farwanya province is a heterogeneous urban area with 234,359 Kuwaitis and 934,934 non-Kuwaitis. Ratio of Kuwaitis to non-Kuwaitis (1 : 2.5) of the selected provinces is consistent with the overall national ratio [10].

Hawali province has eight ambulance stations, with 30 ambulances, 65 EMTs, and 24 paramedics. Al-Farwanya province has nine ambulance stations, with 25 ambulances, 72 EMTs, and 15 paramedics. EMS levels of service are equivalent to North America's Basic and Advanced Life Support levels. Ambulances are staffed with two emergency medical technicians (EMT) per EMS ambulance and one EMT and one paramedic per ALS ambulance.

The EMS protocol of cardiac arrest in Kuwait follows the 2010 AHA CPR guidelines for CPR and defibrillation. However, the protocol states that EMTs cannot remain at the scene beyond one CPR cycle and rhythm analysis, and they must transport the patient to an emergency department while continuing to perform CPR during ambulance transport [9].

Kuwait has a single, centralized dispatch centre for all ambulance services; this is an Arabic-based system and receives calls for EMS and interhospital transportation. For emergency calls, Kuwait follows a European emergency response system. A universal emergency number 1-1-2 has automatic location identification with centralized dispatch for police, fire, and EMS. If medical assistance is needed, the call is forwarded to an EMS call-taker who answers the call, reconfirms the address, and responds by activating the nearest ambulance. Call-related information, including the medical priority dispatch system, is in Arabic.  Table 1 describes key features of Kuwait's EMS.

The average number of calls per year is approximately 90,244, including 9,427 cardiac cases [11].

This study was a retrospective analysis of OHCA cases retrieved from the National EMS archived data between 1 February and 31 December 2017. Collected variables included patient characteristics, event data, and EMS response and interventions. Outcome variables collected were ROSC and survival to hospital discharge.

### 2.2. Participants

Study population included adult patients (>18 years old) with OHCA that activated the National EMS and were treated and transported by EMS. Only patients with cardiac aetiology of OHCA and who were treated in Hawali and Al-Farwanya provinces were included. Patients with noncardiac etiologies of arrest, patients for whom resuscitation was not attempted (decapitation, rigor mortis, and dependent lividity), pregnant women, and unidentified patients (due to difficulty in follow-up) were excluded.

### 2.3. Data Source/Management

Data were collected from various sources using the Utstein Style template. The three data sources were searched retrospectively for OHCA cases: dispatch unit electronic records, audit department archival data, and hospital medical records. The investigator first matched dispatch electronic records with audit department archived data using the patient report form serial number. Patient report form serial number is unique for every case that activates the EMS system. The investigator then matched the audit department archival data with the hospital medical records using the patient civil identification number.

Survival to hospital discharge was collected from hospital records, and prehospital ROSC and Bystander CPR rate were obtained from the patient record form. Dispatch code was collected from dispatch unit electronic records.

### 2.4. Sample Size

Convenient sampling was used in this study. All eligible EMS-treated OHCAs during the study period were included.

### 2.5. Statistical Methods

Statistical analysis was performed using Excel and Statistical Package for Social Sciences (IBM SPSS Version 23, NY, USA). Descriptive analysis was performed to determine distribution, frequency, response time, and age; means and percentages were used to describe and report variables and patient characteristics.

## 3. Results

**Figure 1:**
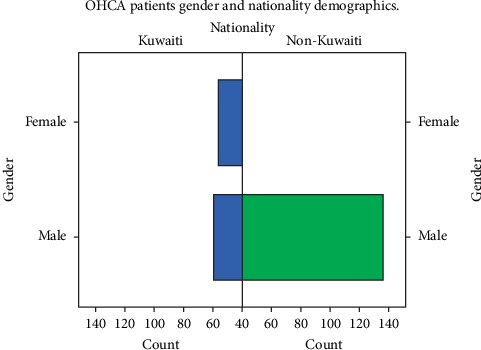
Gender and nationality frequency of OHCA patients in pilot regions of Kuwait, 2017.

**Figure 2:**
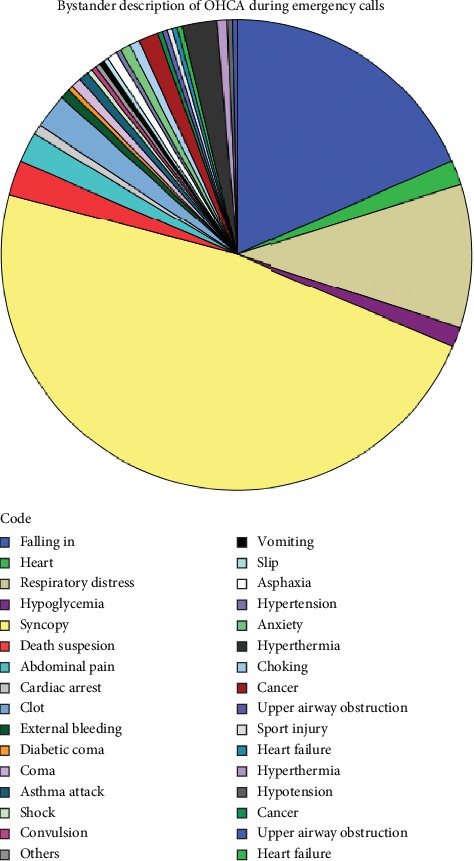
Pie chart of bystander description of OHCA during emergency calls registered by local EMS dispatch unit for pilot regions of Kuwait, 2017.

A total of 332 OHCA cases were treated by EMS during the study period. Only 286 met the inclusion criteria. This corresponded to arrests 13.5 per 100,000 persons. The median age of OHCA patients was 63 years (Table 2). Males outnumbered females by 32% (Figure 1). Only two bystanders recognized the cardiac arrest patients (0.7%) (Figure 2). Sixteen bystanders received dispatcher-assisted CPR, and seven were resuscitated by health professional on scene prior to EMS personnel arrival. Few patients were witnessed (11.5%) with low bystander CPR rate (8.7%). Low shockable rhythm rates (1%) led to rare use of AED by EMS personnel (1%).

And while ten OHCA patients achieved ROSC, only one patient (0.3%) survived to hospital discharge. Additional characteristics and outcomes are presented in Table 2.

## 4. Discussion

This is the first study to report on OHCA survival in Kuwait. Survival to hospital discharge is low (0.3%) when compared to regional (4.8%–8%) [8, 12] and Western countries (10.3%–26.1%) [1, 7].

Several factors may be contributing to this low OHCA survival rate. Few arrests were witnessed in this study. Although most cases occurred at home (76%), low witness rate (11.5%) is evident (Table 2); these findings are different from regional studies [1, 13] but resemble those from US (home 87% and witness rate of 6%) [14]. Lewis et al. (2016) attributed the low witness rate with the low marriage rate of OHCA patients in North Carolina [14]. The present study did not investigate OHCA patients' marital status. Most of OHCA patients were however non-Kuwaiti (Table 2) with increased likelihood that they were single at the time of cardiac arrest. Non-Kuwaiti individuals living in Kuwait predominantly are single men in the workforce, and immigration regulations impose tight restrictions on family immigration [15].

Other reasons for low OHCA survival rates in Kuwait include low early OHCA recognition and poor bystander description of cardiac arrest during EMS calls (Figure 2). Bystander's poor description of cardiac arrest during EMS calls, delayed bystander CPR, and EMS dispatch causing low OHCA survival rates can be improved by concentrating on dispatcher-assisted CPR along with community-based CPR education programme [2]. During the course of this study, only 70 call takers were trained on dispatcher-assisted CPR-SHARE programme. To improve OHCA survival rates in Kuwait, all the call takers should receive similar training.

The study also identifies low bystander CPR rates (8.7%) when compared to the Middle Eastern studies, 30% [3, 12]. Initiating community-CPR-based education programs can be one way to improve bystander CPR rates and subsequently improve OHCA survival [2].

Moreover, low shockable rhythm rate (Table 2) contributes to low OHCA survival rates in regions of Kuwait. OHCA patients with a rhythm other than shockable rhythms have been shown to have a worse prognosis [16].

The local EMS mean response time 9.3 ± 5 minutes is similar to EMS mean response time in other Middle Eastern countries, 10 minutes in the United Arab Emirates [17], and 13 minutes in Saudi Arabia [18]. Basic life support measures were predominantly used during OHCA incidence resuscitation. This is less advanced than EMS practices in the region. Qatar's EMS system resuscitates all OHCA patients' with Advanced Life Support (ALS) and mechanical chest-compression devices by critical-care paramedics [12]. ALS interventions have not, however, been shown to contribute to increased OHCA survival [19]. Focusing on dispatcher-assisted CPR and community-based CPR and consolidating EMS practices are the initial steps to improve OHCA survival in Kuwait.

Demographic characteristics of the study population were similar to regional studies: mean age of patients in this study (61.1 years) was consistent with regional studies (mean age 62.9 years in Saudi Arabia; 69 ± 15.4 years in Lebanon) [4, 13].

Non-Kuwaitis were more likely to experience OHCA. Other regional studies found that non-nationals were more prone to experience OHCA [3, 12]. Moreover, this study identified higher OHCA rates among Kuwaiti females (Figure 1) than previously reported in the current literature [20] Despite similarities of most characteristics, these findings in addition to the low survival rate highlight the need for a national cardiac registry to identify weak links in the system of care and to address gaps in OHCA management.

## 5. Limitations

This study has some limitations. The study sample size is relatively small. Another limitation is that a dispatcher-assisted CPR training program was being carried in Kuwait EMS dispatch unit between February and December 2017; this could have influenced bystander CPR rates in the study regions. Additional limitations are related to missing or incomplete data in this retrospective study. Other limitations include lack of cerebral performance category at hospital discharge and absence of in-hospital intervention evaluation, such as extracorporeal membrane oxygenation, targeted temperature management, and percutaneous coronary intervention,; all could have influenced the results of this study.

## 6. Conclusion

OHCA survival rates in this region of Kuwait are low. Targeted measures such as creating cardiac registry, dispatcher-assisted CPR with ongoing training and quality improvement, and community-based CPR education program are needed to improve the survival rates of OHCA victims.

## Figures and Tables

**Table 1:** Key features of Kuwait emergency medical services.

Call volume in 2017 (incidence/1,000 inhabitants/year)	139,751
Proportion of calls resulting in ambulance	All calls receive an ambulance
Index/dispatch priority tool	All calls are high priority
Manual/electronic use of index	Electronic
Dispatch priority tool	North American Emergency Medical Priority Dispatch (Pro QA version12.1)
Mandatory/optional use of index	Optional
Call takers' certification and education	125 nurses (73%), 47 emergency medical technicians (EMTs) (26%), and 3 paramedics, 1-day intensive course: dispatcher-assisted CPR SHARE course (70)
Approximate number of call takers/24 h shift	25 call takers/shift
Ambulance services (2017 numbers)	114,525 missions 173 ambulances, 3 helicopters
Mean response time	11.3 minutes
First responder system	No first responder system
Public access defibrillation	Not installed
Competence of ambulance providers	Paramedics 150 (25%) EMTs 450 (75%)

**Table 2:** OHCA demographics and outcomes.

Domains (core elements)			21 February–31 December 2017, *N* = 286, *n* = (%)
			*Two-tiered EMS system*
Bystander Recognition			2 (0.7)
Dispatcher-assisted CPR			16 (5.6)
Location		Home	217 (76)
Age (mean ± sd)			61.1 ± 16
Gender		Male	192 (67.1)
		Female	94 (35.9)
Medicalhistory		More than onecardiovascular risk	49 (17.1)
Nationality		Kuwaiti	112 (39.2)
	Non-Kuwaiti		174 (60.8)
Rhythm		Shockable	3 (1)
		Nonshockable	85 (29.7)
Witnessed			33 (11.5)
Basic life support			280 (97.9)
Advanced life support			12 (4.2)
EMS defibrillation			3 (1)
Response time in minutes (mean ± sd)			9.3 ± 5
Bystander CPR			25 (8.7)
ROSC			10 (3.5)
Survival to hospital discharge			1 (0.3)

## Data Availability

The database generated during the current study is summarized and available from the corresponding author on reasonable request.
